# Association between Sleep Quality and C-Reactive Protein: Results from National Health and Nutrition Examination Survey, 2005-2008

**DOI:** 10.1371/journal.pone.0092607

**Published:** 2014-03-24

**Authors:** Rong Liu, Xin Liu, Phyllis C. Zee, Lifang Hou, Zheng Zheng, Yongxiang Wei, Jie Du

**Affiliations:** 1 Beijing An Zhen Hospital, Capital Medical University, The Key Laboratory of Remodeling-related Cardiovascular Diseases, Ministry of Education, Beijing Institute of Heart, Lung and Blood Vessel Diseases, Beijing, China; 2 Mary Ann and J. Milburn Smith Child Health Research Program, Department of Pediatrics, Northwestern University Feinberg School of Medicine and Ann & Robert H. Lurie Children's Hospital of Chicago and Children's Hospital of Chicago Research Center, Chicago, Illinois, United States of America; 3 Department of Neurology and Sleep Medicine Center, Northwestern University Feinberg School of Medicine, Chicago, Illinois, United States of America; 4 Department of Preventive Medicine, Northwestern University Feinberg School of Medicine, Chicago, Illinois, United States of America; 5 Department of Neurobiology, Beijing Institute of Geriatrics, Beijing Xuan Wu Hospital, Capital Medical University, Beijing, China; 6 Department of Otorhinolaryngology, Beijing An Zhen Hospital, Capital Medical University, Beijing, China; Charité Universitätsmedizin Berlin, NeuroCure Clinical Research Center, Germany

## Abstract

**Objective:**

Our objective was to explore the association between poor sleep quality and hs_CRP in an adult U.S. population.

**Methods:**

This study focused on 9,317 participants in the National Health and Nutrition Examination Survey (NHANES) from 2005–2008 who were aged 20–85 years, completed a sleep disorder questionnaire, and had available information on serum hs_CRP. Sleep quality was classified into three categories (good, moderate, poor) based on the responses of participants to the NHANES sleep disorder questionnaire. High CRP was defined as hs-CRP >1 md/dL. Linear regression model was applied to investigate the association between poor sleep quality and log-transformed hs_CRP. And logistic regression model was fitted to evaluate the association between sleep quality and the risk of high CRP.

**Results:**

Females were more likely to report poor sleep quality than males (26% vs. 19%, p<0.0001). Each sleep disorder was significantly associated with increased hs_CRP and correlative to other sleep disorders. In fully-adjusted linear regression model, poor sleep quality was significantly associated with elevated hs_CRP (log transformed) among the overall sample and in females only (β = 0.10, se = 0.03, p<0.01 and β = 0.13, se = 0.04, p<0.01, respectively). In fully-adjusted logistics regression model, poor sleep quality was linked with risk of high CRP(OR: 1.42, 95%CI: 1.15–1.76 in overall sample and OR: 1.59, 95%CI: 1.18–2.14 in females, respectively).

**Conclusion:**

We found that poor sleep quality was independently associated with elevated hs_CRP in females but not in males in a U.S. adult population.

## Introduction

High sensitivity C-reactive protein (hs_CRP) has been the most extensively studied and validated measure of vascular inflammation to date. Since the advent of high sensitivity technology permitting measurement of CRP levels as low as 0.007 mg/dl, compared to the previous detection limits of 3 to 5 mg/dl [1], epidemiologic studies have pointed to CRP as a predictor of both long- and short-term risk of stroke and myocardial infarction in men and women [2], [3]. CRP has been shown to enhance secretion of inflammatory mediators by vascular endothelium [4] and promote uptake of low-density lipoproteins by macrophages in atherosclerotic lesions [5]. These data are indicative of the involvement of CRP in the development of atherosclerotic lesions.

Recently, a series of epidemiologic surveys [6]-[9] discovered that sleep complaints are associated with increased cardiovascular morbidity. Symptoms or diagnosis of insomnia [6], sleep apnea [8],[10] and restless leg syndrome [11] proved to be relative to morbidity and mortality of cardiovascular disease in prospective studies. Although empirical and clinical studies [12], [13] further confirmed a causal relationship between sleep disorders and higher CRP concentrations, results from population studies on sleep and CRP are not consistent [14]–[21]. Most of these studies just focused on only one sleep disorder but ignored others. To date, little population study has evaluated the association between sleep and CRP with sleep quality as sleep index to cover as many different complaints/disorders as possible. Sleep quality [22] is a comprehensive index that includes symptoms regarding insomnia, day time sleepiness, sleep deficiency, sleep apnea, and restless legs syndrome. It, in fact, reflects the nature and magnitude of sleep loss. Given that CRP concentration is influenced by a series of factors, like smoking, alcohol consumption, medication use, obesity, and race as well as gender [23], even sleep loss has been proven to lead to increased CRP in empirical study, though whether a relationship between sleep quality and CRP truly exists in the real world remains uncertain. Taking advantage of the National Health and Nutrition Examination Survey (NHANES) design, we aimed to investigate whether poor sleep quality is independently associated with CRP using a representative multiracial sample of U.S. adults.

## Methods

### Study population

The data for this study was derived from the NHANES conducted from 2005–06 and 2007–08. A detailed description of NHANES study design and methods are available elsewhere [24], [25]. Briefly,the NHANES survey included a stratified multistage probability sample, representative of the civilian non-institutionalized U.S. population. Selection was based on counties, blocks, households and individuals within households, and included oversampling of non-Hispanic black and Mexican Americans in order to provide stable estimates of these groups. We restricted our study sample to participants aged greater than 20 years. Questions on sleep were first included in the NHANES 2005–2006 and continued in NHANES 2007–2008. Of the 10,480 participants with information on sleep and hs_CRP, after excluding those with missing data on sleep variables, body mass index and hs_CRP (n = 800), those with outlier values for hs_CRP (n = 8) and females with pregnancy (n = 355), 9,317 subjects were left for the following analysis. The NHANES protocol was approved by National Center for Health Statistics(NCHS)Ethics Review Board [26], and written informed consent was obtained from all participants.

### Measurements

Hs_CRP were quantified using latex-enhanced nephelometry and a Behring Nephelometer II Analyzer (Immunology Division, Department of Laboratory Medicine, University of Washington Medical Center, Seattle, Washington). Due to the skewed distribution, log-transformed hs_CRP were used in all analyses. Additionally, high CRP was defined as >1 mg/dL, consistent with American Heart Association/Certers for Disease Control& Prevention(AHA/CDC) guardlines for identifying subjects with moderate/high risk of cardiovascular disease [27].

A sleep quality index was derived from modified criteria by Pooja B. et al [22]. Sleep quality was classified as “good”, “moderate” and “poor” according to participant response to the eight questionnaire items for past 12 months on sleep quality as follows: “ (1)How often have trouble falling asleep; (2) How often wake up during the night and having trouble getting back to sleep; (3) How often wake up too early in the morning and being unable to get back to sleep; (4) How often feel unrested during the day, no matter how many hours of sleep were obtained; (5) How often feel excessively or overly sleepy during the day;(6) How often did you not get enough sleep; (7) How often have leg jerks while sleeping; (8) How often have legs cramp while sleeping.” Participants who answered “almost always” (16–30 times a month) to any of the eight items above were defined as having poor sleep quality; else participants who answered “often”(5–15 times a month) were defined as having moderate sleep quality; and all others were defined as having good sleep quality.

Body mass index (BMI) was classified into underweight (BMI<18.5), normal weight (18.5≤BMI<25), overweight (25≤BMI<30), and obese (BMI≥30) according to WHO definitions. We combined underweight and normal weight into one group in the later analyses because of the small sample size (n = 53 males and n = 95 females) of underweight participants.

Information on age, gender, race/ethnicity, smoking status, alcohol intake, education level, estrogen/progestin use, and statin use was obtained using a standardized questionnaire during a home interview. Smoking status was determined by responses to the two items: (1) “smoked at least 100 cigarettes in life?” (2) “Do you now smoke cigarettes?” Participants who answered “yes” to the first item and “everyday/some days” to the second were defined as smokers. Others were defined as non-smokers. Alcohol intake was dichotomized as drinkers and non-drinkers based on response to the following item: “had at least 12 alcohol drinks/lifetime”.

### Statistical Methods

To account for the complex survey design and to obtain results that would be generalizable to a U.S. non-institutionalized population, standard errors of the means and percentiles were weighted using NHANES 4-year calculated examination sample weights (MEC4YR = 1/2*wtmec2yr) to produce a national estimate [28]. We first estimated the correlation between each components of sleep quality and hs-CRP concentration, separately. Then we assessed the association of all eight components of sleep quality and hs-CRP with regression model. Finally, we assessed whether a combined sleep quality was associated with log-transformed hs_CRP. Logistic regression model was further conducted to evaluate the association of sleep quality with risk of high CRP. We used self-reported health status as a proxy for several health conditions that might be linked with chronic inflammation. Model 1 was adjusted for age, race, gender, education smoking status, alcohol intake, estrogen/progestin and statin use as well as self-reported health status. Model 2 was further adjusted for BMI status in addition to the other covariates included in Model 1. Because of the known gender difference in CRP concentration [29], all analyses were further stratified by gender. We used SAS software (version 9.2, SAS Institute, Inc., Cary, North Carolina) for all analyses. P<0.05 was considered to be statistically significant. All reported p-values were two-sided.

## Results

Table 1 presents the anthropometric and demographic characteristics of the study population. The average age of the population was 47.1 years, the average BMI was 28.6, and 51% of the participants were female. A majority of the participants were non-Hispanic Whites (71%) and alcohol drinkers (73%). Fifty-six percent of subjects achieved an education level above high school and only 24% were current smokers. Females who reported using estrogen/progestin accounted for 13% of the population, and only 15% of the population reported using statins. Overall, we found significant gender differences in race, smoking, alcohol drinking and age (p<0.0001).

**Table 1:** Selected adult population (20–85 yrs old) characteristics: NHANES, 2005–2008.

	Total	Male	Female	P -value
		Mean (se)^a^		
Age(yrs)	47.1(0.4)	46.1(0.5)	48.0(0.5)	<0.0001
BMI(kg/m^2^)	28.6(0.1)	28.6(0.2)	28.5(0.2)	0.7463
		N (%^b^)		
Females(%)	4627(51)	––	––	
Race/Ethnicity				
Non-Hispanic White	4590(71)	2386(71)	2222(72)	<0.0001
Non-Hispanic Black	1934(11)	949(10)	985(11)	
Mexican American	1712(8)	863(9)	849(7)	
Other	1081(10)	510(10)	571(10)	
Education				0.0396
Below high school	2723(19)	1423(20)	1300(18)	
High school	2262(25)	1161(26)	1101(24)	
Above high school	4332(56)	2106(54)	2226(58)	
Smoking	2115(24)	1229(23)	886(20)	<0.0001
Drinking	6256(73)	3403(78)	2853(69)	<0.0001
Estrogen/progestin use	456(7)	––	456(13)	––
Statin use	1589(15)	812(15)	777(15)	0.8973
BMI status^c^				
Underweight	148(1)	53(1)	95(2)	
Normal weight	2608(31)	1222(26)	1386(35)	
Overweight	3220(34)	1882(41)	1974(40)	
Obese	3341(34)	1533(32)	1450(35)	<0.0001

Student's t-test was used to compare continuous variables, and x^2^ was used to compare categorical variables between males and females. ^a^weighted mean. ^b^weighted percentage. ^c^underweight (BMI<18.5); normal weight (18.5≤BMI<25); overweight (25≤BMI<30); obese (BMI≥30).

Table 2 outlines the profile of sleep quality components stratified by gender. Each component was rated according to the frequency reported by the participants. Combined sleep quality was defined in terms of the highest frequency of components. Females were more likely to report sleep disorders for each component. And more than one fourth of females reported suffering 16-30 times per month from at least one sleep disorders while only 19.6% males experienced poor sleep quality. The sleep-CRP relationship in terms of each component of sleep quality was summarized in Table 3. The component scores of sleep quality was significantly associated with hs-CRP, which suggested a dose relationship between the frequency of sleep problem with hs-CRP(all p<0.05), even after adjustment for age, gender, smoking, drinking and estrogen or statin use. However, when all the components were considered simultaneously in regression model, the significance for all components disappeared (data not shown). From Spearman coefficient matrix (Table 4) we found that each component was significantly associated to other components with coefficient ranging from 0.17–0.65(p<0.001 for all components).

**Table 2:** Profile of sleep quality components in American Adults:NHANES 2005–2008.

Components	Score*	Total(%)	Male(%)	Female(%)	P value#
Having trouble falling asleep	1	72.4	87.0	77.9	
	2	10.0	7.5	12.5	
	3	7.6	5.5	9.6	<0.0001
Waking up during night	1	29.1	83.9	74.9	
	2	13.2	10.4	15.9	
	3	7.5	5.7	9.2	<0.0001
Early wake-up in morning	1	82.9	84.5	81.4	
	2	10.9	10.0	11.8	
	3	6.2	5.5	6.8	0.0029
Feeling unrested	1	72.1	77.8	59.7	
	2	16.7	14.3	18.9	
	3	10.2	7.9	12.4	<0.0001
Feeling overly sleepy	1	81.6	84.7	78.7	
	2	12.5	10.2	14.6	
	3	5.9	5.1	6.7	<0.0001
Feeling not enough sleep	1	73.7	85.2	71.1	
	2	16.1	9.1	17.8	
	3	10.2	5.7	11.1	<0.0001
Leg jerks	1	93.7	97.2	93.0	
	2	3.7	1.7	4.0	
	3	2.6	1.1	3.0	0.1078
Leg cramp	1	94.0	94.7	92.4	
	2	4.2	2.9	5.4	
	3	1.8	2.4	2.2	<0.0001
sleep quality**	Good	49.5	54.8	44.5	
	Moderate	27.6	25.7	29.4	
	Poor	22.9	19.5	26.1	<0.0001

*score: 1 =  Never-4 times/month; 2 = 5–15 times/month; 3 = 16–30 times/month.

**Poor: at least one component meet scale 3; Moderate: at least one component meet scale 2; Good: no component meet scale 2 or 3.

# chi-square test for frequency between genders.

**Table 3:** Correlation between score of sleep quality components and hs-CRP in American adults: NHANES 2005-2008.

	Falling sleep trouble	Waking up during night	Early wake up in morning	Feeling unrested	Feeling overly sleepy	Feeling not enough sleep	Leg jerk in sleep	Leg cramp in sleep
Log(hs_CRP)	0.045	0.040	0.022	0.048	0.036	0.037	0.034	0.050
*P*	<0.0001	0.0001	0.0373	<0.0001	<0.0001	0.0004	0.0011	<0.0001

Adjusted the effects of age, gender, estrogen use, statin use, smoking and drinking.

**Table 4:** Spearman coefficients among sleep quality components scale in American adults: NHANES 2005–2008.

	Falling sleep trouble	Waking up during night	Early wake up in morning	Feeling unrested	Feeling overly sleepy	Feeling not enough sleep	Leg jerk in sleep	Leg crampin sleep
Falling sleep trouble	1.00							
Waking up during night	0.54	1.00						
Early wake up in morning	0.41	0.60	1.00					
Feeling unrested	0.37	0.37	0.35	1.00				
Feeling overly sleepy	0.31	0.33	0.29	0.65	1.00			
Feeling not enough sleep	0.42	0.42	0.39	0.56	0.51	1.00		
Leg jerk in sleep	0.20	0.18	0.15	0.19	0.17	0.15	1.00	
Leg cramp in sleep	0.17	0.19	0.18	0.18	0.18	0.15	0.36	1.00

P<0.001 for all coefficients of two different components.

In the multiple regression analyses of the overall population, hs-CRP was significantly associated with age, gender, race/ethnicity, education, smoking, drinking, estrogen/progestin use, statin use, health status and sleep quality independent of obesity status (Table S1). Table 5 summarizes association of sleep quality with hs-CRP from linear regression and logistic models. Before further adjustment for BMI, hs_CRP (log transformed) was 0.19 units higher for subjects with poor sleep than for those with good sleep (p<0.0001). After adjustment for BMI, the association between poor sleep quality and hs_CRP was attenuated by almost 50%: poor sleep quality increased 0.10 hs_CRP units (p<0.01). When we stratified the analyses by gender, this association remained significant in females but not in males (β = 0.26, se = 0.05, p<0.0001, before adjustment; β = 0.13, se = 0.04, p<0.01, after adjustment). Likely, in fully-adjusted logistic regression models, poor sleep quality was associated with increased risk of high CRP in total population (odds ratio (OR): 1.46, 95% confidence interval (95%CI): 1.15-1.72.). However, we further found that poor sleep quality was associated with the risk of high CRP only in females (OR:1.59, 95%CI:1.18–2.14) but not in males(Table 5). As expected, only in females, sleep quality scores were significantly associated with hs-CRP.

**Table 5:** Association of sleep quality with hs-CRP stratified by gender and race/ethnicity.

Sample	Sleep quality	Linearbeta	regression Model (se)	Logisticsodds ratio	Model (95%CI)
		Model 1	Model 2	Model 1	Model 2
Total	Good	Reference	Reference	1.00	1.00
	Moderate	0.03(0.04)	0.03(0.04)	1.10(0.88–1.37)	1.10(0.87–1.87)
	Poor	0.16(0.04)^&^	0.10(0.03)^#^	1.54(1.26–1.88)^&^	1.42(1.15–1.76)^#^
	*P* trend	0.0002	0.0079	<0.0001	<0.0001
Males	Good	Reference	Reference	1.00	1.00
	Moderate	0.00(0.06)	0.00(0.05)	1.22(0.81–1.84)	1.22(0.81–1.85)
	Poor	0.07(0.04)	0.05(0.04)	1.13(0.80–1.59)	1.11(0.78–1.57)
	*P* trend	0.44	0.37	0.37	0.37
Females	Good	Reference	Reference	1.00	1.00
	Moderate	0.07(0.06)	0.07(0.05)	1.06(0.86–1.34)	1.11(0.78–1.57)
	Poor	0.26(0.05)^&^	0.13(0.04)^&^	1.77(1.35–2.33)^&^	1.59(1.18–2.14)^#^
	*P* trend	0.0003	0.0069	0.0001	0.0001

Model 1, adjusted for sex, age, smoking, drinking, estrogen/progestin use, statin use, education status, race/ethnicity, self-reported health status. Model 2, model 1 further adjusted for BMI. ^&^p<0.001, ^#^p<0.01.

## Discussion

This is the first population study of the association between sleep quality and hs_CRP. In this large, cross-sectional study of nationally representative sample of adult American population, we found that females were more likely to report poor sleep quality than males. Overall, poor sleep quality was independently associated with higher hs_CRP. Such association remained significant and became even stronger in females but was absent in males.

NHANES initiated the sleep disorder survey from 2005–2006 [24] and continued the sleep survey using the same questionnaire in 2007–2008 [25]. The questions regarding sleep quality included symptoms/complaints of insomnia, daytime sleepiness and restless leg syndrome, which are usually comorbid with short sleep time and OSA. Sleep loss is the primary impairment underlying most sleep disorders [30]. Since the close correlation among these sleep disorders may lead to failure in detecting the association of sole sleep disorder and CRP, as we found in this study (Table 3), a combined index should be more representative than separate components in sleep quality evaluation. Hence, to represent as many sleep disorders (sleep curtailment, sleep apnea, restless legs syndrome) as possible, we defined the sleep quality index using modified previous criteria [22] as a proxy of sleep loss according to the participants' responses to sleep quality questions.

Although this study is the first to evaluate the association between sleep quality and CRP in a large population, earlier experimental studies on the impact of sleep restriction and sleep disorders on inflammatory and immunologic parameters have been reported. Both acute total and short-term partial sleep deprivation were found to lead to elevated hs_CRP concentrations [13]. Neurobehavioral signs and symptoms of fatigue and sleepiness were induced in subjects undergoing sleep loss [31], [32]. Moreover, IL-6—CRP control factor was increased after sleep deprivation for 24 hours [33] and 88 hours [34]. These results also support the hypothesis that one way in which CRP becomes elevated in patients with untreated OSA is through the deprivation of restorative sleep. However, results from epidemiologic studies regarding sleep disorders and CRP are not consistent. A study of 316 Japanese men showed that sleep disordered breathing was associated with CRP. Another study found that severe periodic leg movements were linked with increased CRP in restless leg syndrome [15]. The Wisconsin Sleep Cohort study [14] and the latest large population study using data from NHANES [16] yet failed to detect any significant association of short sleep duration or sleep apnea with CRP. Some studies with separate analysis of gender found disparity in sleep-CRP relationship. In a cross-sectional analysis of 6465 participants aged 50–99 years from the English Longitudinal Study of Ageing [21], long sleep duration was found associated with increased CRP only in men but not in women. Miller, A. et al's discovery [4] also suggested gender difference in linkage between short sleep and CRP, in which only women who slept short time had high CRP. Other studies [19], [20] on sleep duration, either exploring sleep-CRP association just in whole sample or including only one gender, contributed limited information on gender difference. The Hunt study of 8547 men and non-pregnant women also did not support a significant association between insomnia and CRP [17]. We presume that the reason for failure to detect an association between sleep disorders and CRP in some population studies might rest in an incomplete estimation of the sleep disorders, which could be a result of just focusing on one type of sleep disorder/problem but neglecting others. The present study design addressed this issue by defining the sleep quality measurement to reflect most frequently studied sleep disorders/problems such as insomnia, sleep fragmentation, sleep apnea and restless legs.

The linkage of poor sleep quality with increased CRP may also be mediated by adiposity. Increased BMI has been shown to be susceptible to cytokinemia due to the synthesis and release of IL-6 from adipose tissue [35]. The Sleep Debt Study [36], [37] by Spiegel et al. found that experimentally induced partial sleep deprivation could decrease insulin sensitivity and boost appetite. Many prospective epidemiologic investigations have found short sleep duration to be associated with weight gain in both children and adults [38]–[42]. These findings suggest that weight status should be taken into account when estimating the relationship between sleep disorders and CRP. Our results show that BMI partially mediated the strong association between poor sleep quality and CRP, and BMI alone explained a much larger variance of CRP compared to other factors taken together in both genders.

The gender difference in the association between poor sleep quality and CRP was thought to be attributed to the lower prevalence of poor sleep in males than in females. Our results show clear differences between males and females in the prevalence of insomnia, daytime sleepiness and feeling unrested, symptoms of restless leg syndrome and snoring/snorting (data not shown). Males were more likely to report daytime sleepiness/feeling unrested and snoring/snorting, while females were more vulnerable to insomnia, sleep disturbances or restless legs. It is possible that the sleep quality index failed to exactly represent sleep disorders in males. A more specific index of sleep problems for males is needed for future study.

The strengths of our study include its large sample size and wide age range (20–85 yrs). The sampling scheme of the NHANES allowed for better estimates and control for various race/ethnicity backgrounds, behavioral and medication factors. We selected covariates according to previous evidence relative to hs_CRP and sleep. However, given that this was a cross-sectional analysis, our study could not determine a causal relationship from sleep to hs_CRP. Our results also should be interpreted in light of another limitation. Physical actively is well known to benefit CRP concentration [43]. However, in this study, due to the different questionnaires regarding physical activity that were applied in the 2005–2006 and 2007–2008 surveys, we could not define the unique surrogate to physical activity level or control for it when estimating the association between sleep quality and CRP. Assuming that physical activity improves BMI status, the inclusion of BMI in the regression model could partially offset the absence of a physical activity factor.

In summary, we found that poor sleep quality was independently associated with higher hs_CRP in females, indicating that inflammation may be operative in conditions such from sleep complaints to atherosclerotic vascular disease. If further longitudinal studies confirm the causal relationship of poor sleep with high hs_CRP, new approaches to lower the risk for cardiovascular disease in individuals with poor sleep quality should be evaluated.

## Supporting Information

Table S1 Multiple liner regression coefficients for the association of poor sleep with hs_CRP in American adults.
(DOCX)
