# Supplementary material for: Association between Sleep Quality and C-Reactive Protein: Results from National Health and Nutrition Examination Survey, 2005-2008
Source: PLoS One. 2014 Mar 24;9(3):e92607. doi: 10.1371/journal.pone.0092607 (PMC3963926; doi:10.1371/journal.pone.0092607)
Supplement: Table S1 — Multiple liner regression coefficients for the association of poor sleep with hs_CRP in American adults. (DOCX) [file pone.0092607.s001.docx]

| Table S1. Multiple liner regression coefficients for the association of poor sleep with hs_CRP in American adults. | | | | | | |
| --- | --- | --- | --- | --- | --- | --- |
| Dependent variable Total Males Females | | | | | | |
| log hs_CRP | Model 1 | Model 2 | Model 1 | Model 2 | Model 1 | Model 2 |
|  | Β(se) | Β(se) | Β(se) | Β(se) | Β(se) | Β(se) |
| Age(10 yrs) | 0.12(0.01)^&^ | 0.12(0.01)^&^ | 0.16(0.01)^&^ | 0.15(0.01)^&^ | 0.09(0.01)^&^ | 0.09(0.1)^&^ |
| Gender(female) | 0.15((0.03)^&^ | 0.20(0.03)^&^ | --- | --- | --- | --- |
| Race/Ethnicity White | Reference | Reference | Reference | Reference | Reference | Reference |
| Black | 0.28(0.04)^&^ | 0.15(0.03)^&^ | 0.16(0.05)^#^ | 0.13(0.04)^#^ | 0.37(0.06)^&^ | 0.11(0.05) |
| Mexican | 0.27(0.06)^&^ | 0.20(0.05)^&^ | 0.20(0.06)^#^ | 0.17(0.06)^#^ | 0.36(0.08)^#^ | 0.23(0.07) |
| Other | -0.10(0.07) | 0.06(0.06) | 0.04(0.10) | 0.10(0.08) | -0.23(0.08)^&^ | -0.11(0.06) |
| BMI rank Normal/Underweight | --- | Reference | --- | Reference | --- | Reference |
| Overweight |  | 0.62(0.04)^&^ |  | 0.46(0.06)^&^ |  | 0.73(0.04)^&^ |
| Obese |  | 1.37(0.03)^&^ |  | 1.19(0.05)^&^ |  | 1.59(0.05)^&^ |
| Education Above high school | Reference | reference | Reference | Reference | Reference | Reference |
| High school | 0.13(0.03)^#^ | 0.06(0.03)^*^ | 0.15(0.05)^#^ | 0.12(0.05)^*^ | 0.14(0.06)^*^ | 0.04(0.05) |
| Below high school | 0.11(0.03)^#^ | 0.08(0.04)^*^ | 0.05(0.05) | 0.06(0.05) | 0.19(0.05)^&^ | 0.08(0.04) |
| Smoking | 0.21(0.04)^&^ | 0.35(0.03)^&^ | 0.29(0.05)^&^ | 0.40(0.05)^&^ | 0.14(0.05)^*^ | 0.27(0.05)^&^ |
| Drinking | -0.16(0.03)^&^ | -0.10(0.03)^#^ | -0.09(0.04)^*^ | -0.07(0.04) | -0.23(0.05)^&^ | -0.15(0.04)^#^ |
| Estrogen/progestin use | 0.71(0.08)^&^ | 0.87(0.07)^&^ | -- | -- | 0.69(0.08)^&^ | 0.87(0.07)^&^ |
| Statin use | -0.02(0.05) | -0.15(0.05)^#^ | 0.04(0.05) | 0.22(0.05)^&^ | -0.08(0.07) | -0.07(0.07) |
| Sleep quality Good | Reference | Reference | Reference | Reference | Reference | Reference |
| Moderate | 0.03(0.04) | 0.03(0.04) | 0.00(0.06) | 0.00(0.05) | 0.07(0.06) | 0.07(0.05) |
| Poor | 0.16(0.04)^&^ | 0.10(0.03)^#^ | 0.07(0.04) | 0.05(0.04) | 0.26(0.05)^&^ | 0.13(0.04)^&^ |
| R^2^ | 0.07 | 0.26 | 0.07 | 0.20 | 0.06 | 0.32 |
| Model 1, adjusted for sex, age, smoking, drinking, estrogen/progestin use, statin use, education status, race/ethnicity, self-reported health status. Model 2, model 1 further adjusted for BMI. ^&^p<0.001, ^#^p<0.01, *p<0.05 | | | | | | |
